# Nimbolide Targets Multiple Signalling Pathways to Reduce Neuroinflammation in BV-2 Microglia

**DOI:** 10.1007/s12035-023-03410-y

**Published:** 2023-06-14

**Authors:** Folashade O. Katola, Olumayokun A. Olajide

**Affiliations:** 1 Department of Pharmacy, School of Applied Sciences, University of Huddersfield, Queensgate, Huddersfield, HD1 3DH UK; 2 University of Texas Southwestern Medical Center, Dallas, TX 75390-9072 USA

**Keywords:** Nimbolide, Neuroinflammation, NF-κB, MAPK, Nrf2, SIRT-1

## Abstract

Nimbolide, a limonoid compound found in the neem plant, was investigated for effects on neuroinflammation in BV-2 microglia activated with lipopolysaccharide (LPS). Cultured BV-2 cells were treated with nimbolide (125, 250 and 500 nM) followed by stimulation with LPS (100 ng/ml). Results showed that nimbolide caused a significant reduction in the levels of TNFα, IL-6, IFNγ, NO/iNOS and PGE_2_/COX-2 in LPS-activated BV-2 cells. Further experiments revealed that LPS-induced increased expression of phospho-p65 and phospho-IκBα proteins were reduced in the presence of nimbolide. Also, LPS-induced NF-κB acetylation, increased binding to consensus sites and transactivation, as well as phosphorylation of p38 and JNK MAPKs were reduced by nimbolide. Reduction of cellular ROS generation by nimbolide was accompanied by a reduction in gp91phox protein levels, while antioxidant effects were also observed through elevation in protein levels of HO-1 and NQO-1. It was observed that treatment of BV-2 microglia with nimbolide resulted in reduced levels of cytoplasmic Nrf2, which was accompanied by increased levels in the nucleus. Furthermore, treatment with this compound resulted in increased binding of Nrf2 to antioxidant responsive element (ARE) consensus sites accompanied by enhanced ARE luciferase activity. Knockdown experiments revealed a loss of anti-inflammatory activity by nimbolide in cells transfected with Nrf2 siRNA. Treatment with nimbolide resulted in nuclear accumulation of SIRT-1, while siRNA knockdown of SIRT-1 resulted in the reversal of anti-inflammatory activity of nimbolide. It is proposed that nimbolide reduces neuroinflammation in BV-2 microglia through mechanisms resulting in dual inhibition of NF-κB and MAPK pathways. It is also proposed that activation of Nrf2 antioxidant mechanisms may be contributing to its anti-inflammatory activity.

**Graphical Abstract:**

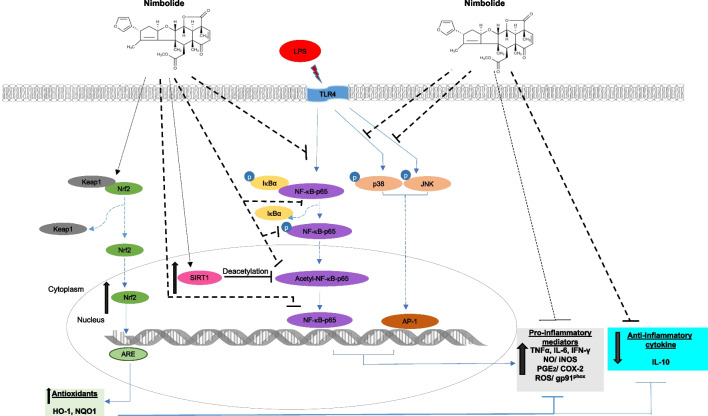

## Introduction

Accumulating evidence continues to implicate microglia-mediated neuroinflammation in the pathogenesis of neurodegenerative disorders, such as Alzheimer’s disease, Parkinson’s disease, muscular amyotrophic lateral sclerosis and multiple sclerosis [1]. Microglia-mediated neuroinflammation has also been linked to the neurological dysfunctions in parasitic diseases such as cerebral malaria [2], African trypanosomiasis [3], viral diseases such as viral encephalitis [4] and HIV [5], as well as early stages of neuropathic pain [6–8]. Consequently, inhibition of neuroinflammation remains a viable strategy in the search for novel neuroprotective drugs.

Multiple cellular signalling processes have been linked to microglia-mediated neuroinflammation; evidence suggests that targeting critical steps involved in these signalling pathways is an essential strategy for the discovery of novel compounds for treating neurodegenerative and other CNS conditions.

The NF-κB transcription factor remains one of the most investigated drug targets for suppressing microglia-mediated neuroinflammation. NF-κB is principally responsible for the regulation of genes associated with critical processes involved in inflammatory responses. A considerable body of evidence suggests that NF-κB activation in the CNS results in multi-cellular processes and gene transactivation, which are implicated in the initiation and progression of neurodegenerative diseases [9]. Following microglia activation, NF-κB is translocated to the nucleus to induce the transactivation of pro-inflammatory genes and neurotoxic factors, which promote neurodegeneration [9]. This has led to several studies to identify novel inhibitors of NF-κB for suppressing neuroinflammation and neurodegeneration [10–12].

The mitogen-activated protein kinases (MAPKs) include c-Jun NH2-terminal kinase (JNK), p38 MAPK and extracellular signal-regulated kinase. Of the MAPKs, the p38 MAPK is known to contribute to microglia-mediated neuroinflammation [13–15]. Studies have suggested that deficiency of MAPK-activated protein kinase 2 (an upstream kinase of p38) resulted in the inhibition of pro-inflammatory mediator release and neurotoxicity [16]. Consequently, targeting microglia MAPK activation is also a valuable pharmacological strategy for inhibiting neuroinflammation.

Oxidative stress is one of the main contributors to the pathogenesis of neurodegenerative and neurological disorders [17, 18]. In order to combat oxidative stress, an antioxidant defence system is activated through the regulatory functions of the nuclear factor erythroid 2-related factor 2 (Nrf2). Studies have suggested that crosstalk between Nrf2 and NF-κB could result in anti-inflammatory activity by inhibiting oxidative stress-induced NF-κB activation [19–21]. Studies have also shown that Nrf2 activation may be contributing to the inhibition of neuroinflammation by some compounds [22–25].

SIRT-1 is a deacetylase which has been widely reported to produce anti-inflammatory properties [26–29]. Studies have further established that SIRT-1 is anti-inflammatory by suppressing NF-κB through the deacetylation of the RelA/p65 subunit of NF-κB at Lys310 [30–32]. Consequently, activation of SIRT-1 could be a mechanism through which compounds possibly inhibit neuroinflammation and thus provides a target for pharmacological modulation.

**Fig. 1:**
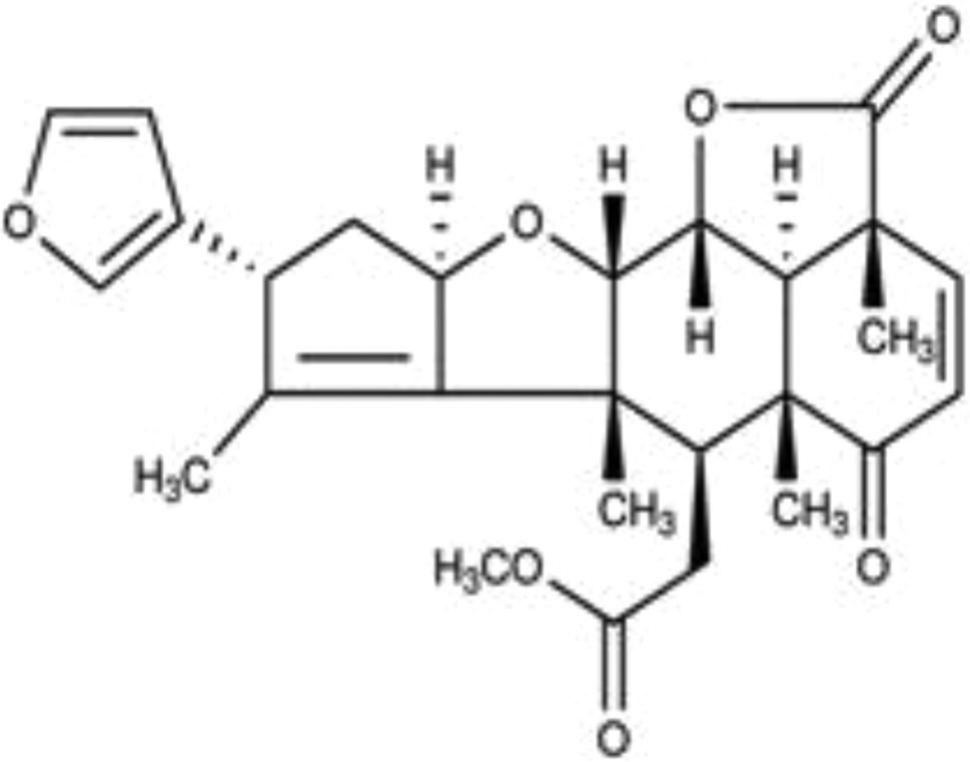
Chemical structure of nimbolide

Nimbolide (Fig. 1) is a tetranortriterpenoid compound found in *the Azadirachta indica* (neem) plant. This compound has been widely investigated as a natural anticancer product [33–36]. Nimbolide has also been reported to produce antiplasmodial activity against *Plasmodium falciparum* in vitro [37]. Studies reported by Diddi et al. showed that nimbolide produced anti-inflammatory and anti-fibrotic effects in bleomycin-induced scleroderma [38]. Further evidence on the anti-inflammatory activity of this compound was provided in a study that showed its effects in reducing inflammatory mediator release in lipopolysaccharide (LPS)-stimulated RAW264.7 macrophages, and in dextran sulphate sodium–induced acute colitis [39]. However, it is not clear if nimbolide provides neuroprotection through the inhibition of signalling pathways in microglia-mediated neuroinflammation.

In this study, we investigated the effects of nimbolide on LPS-induced neuroinflammation in BV-2 microglia and explored the potential molecular mechanisms involved in its anti-inflammatory activity.

## Materials and Methods

### Drugs and Chemicals

Nimbolide was purchased from Sigma, dissolved in dimethylsulfoxide to a concentration of 0.05M, and aliquots were stored at −20°C. LPS derived from *Salmonella enterica* serotype Typhimurium was purchased from Caltag Medsystems (UK).

### Cell Culture

BV-2 mouse microglia cell line (ICLCATL03001) was purchased from Interlab Cell Line Collection (Banca Biologica e Cell Factory, Italy) and cultured in RPMI medium supplemented with 10% foetal bovine serum.

### Determination of BV-2 Microglia Viability

Cultured BV-2 cells were incubated with or without LPS (100 ng/ml) in the presence of nimbolide (125, 250 and 500 nM) for 24 h. Thereafter, XTT/PMS solution (25 μl) was added to the cells in 100 μl of culture medium and incubated for a further 2 h. Cell viability was determined by measuring absorbance 450nm.

In separate experiments, LDH release by cells was determined by transferring 50 μl of cell supernatants into a 96-well plate, followed by the addition of 50 μl of CytoTox 96® reagent (Promega) and incubation in the dark at room temperature for 30 min.

### Production of Pro- and Anti-inflammatory Cytokines, NO and PGE_2_

Cultured BV-2 microglia were treated with nimbolide (125, 250 and 500 nM) for 30 min, followed by stimulation with LPS (100 ng/ml) for a further 24 h. Levels of pro-inflammatory (TNFα, IL-6 and IFNγ) and anti-inflammatory (IL-10) cytokines in culture supernatants were measured using mouse ELISA kits (Biolegend), according to the manufacturer’s instructions. Production of nitrite (as a measure of NO release) in culture supernatants was determined using the Griess assay kit (Promega, UK), according to the manufacturer’s instructions. PGE_2_ levels were determined with a PGE_2_ enzyme immunoassay kit (Arbor Assays, USA), according to the manufacturer’s instructions.

### Cellular Generation of Reactive Oxygen Species

Generation of intracellular reactive oxygen species (ROS) in BV-2 microglia was evaluated using 2′,7′-dichlorofluorescin diacetate (DCFDA)-cellular reactive oxygen species detection assay kit (Abcam). BV-2 microglia in 96-well plates were washed and stained with 20 μM DCFDA for 45 min at 37°C. Cells were then washed and pre-treated with nimbolide (125, 250 and 500 nM) for 30 min, followed by stimulation with LPS (100 ng/ml) for 24 h. ROS generation was measured using microplate fluorescence detection of in a POLARstar Optima microplate reader (BMG Labtech).

Live imaging of ROS generation in BV-2 was carried out using Image-iT^TM^ live Green Reactive Oxygen Species Detection Kit (ThermoFisher). Cultured BV-2 cells were washed with Hanks’ Balanced Salt Solution, and stained with DCFDA for 45 min at 37°C. This was followed by treatment with nimbolide (125, 250 and 500 nM) for 30 min, and stimulation with LPS (100 ng/ml) for a further 24 h. Fluorescence images were captured using EVOS FLoid cell imaging station.

### Immunoblotting

Equal amounts of protein (20–30μg) from samples were subjected to SDS-PAGE under reducing conditions. This was followed by protein transfer to polyvinylidene fluoride membrane (Millipore), which was then blocked for 1 h at room temperature, washed with Tris-buffered saline + 0.1% Tween 20 (TBS-T) and incubated overnight at 4°C with primary antibodies. Primary antibodies used were rabbit anti-iNOS (Cell signalling, 1:1000), rabbit anti-COX-2 (Cell signalling, 1:1000), rabbit anti-phospho-p65 (Cell signalling, 1:1000), κ rabbit anti-phospho-IκBα (Abcam, 1:5000), rabbit anti-phospho-JNK (Cell signalling, 1:1000), rabbit anti-total-JNK (Cell signalling, 1:1000), rabbit anti-phospho-p38α (Cell signalling, 1:1000), rabbit anti-total-p38 (Santa Cruz, 1:500), rabbit anti-gp91phox (Abcam, 1:5000), rabbit anti-Keap1 (Santa Cruz, 1:500), mouse anti-Nrf2 (Santa Cruz, 1:500), rabbit anti-HO1 (Santa Cruz, 1:500), rabbit anti-NQO1 (Santa Cruz, 1:500), rabbit anti-acetyl-p65 (Cell Signalling, 1:1000), rabbit anti-SIRT1 (Santa Cruz, 1:500), rabbit anti-actin (Sigma, 1:1000) and rabbit anti-lamin B1 (Santa Cruz, 1:500). Then, the membrane was washed with TBS-T followed by incubation with Alexa Fluor 680 goat anti-rabbit secondary antibody (1:10000; Life Technologies, UK) at room temperature for 1 h. Blots were detected using LI-COR Odyssey Imager. All western blot experiments were carried out at least three times.

### Transcription Factor Assays

An ELISA-based DNA binding assay was used to determine the effects of nimbolide on the DNA binding of the transcription factors NF-κB and Nrf2. Nuclear extracts were obtained from BV-2 microglia treated with nimbolide (125, 250 and 500 nM) for 24 h. These extracts were then used for DNA binding assays using TransAM Nrf2 transcription factor ELISA Kit (Activ Motif, Belgium), containing a 96-well plate to which an oligonucleotide containing the antioxidant responsive element (ARE) consensus binding site (5′-GTCACAGTGACTCAGCAGAATCTG-3′) was immobilised.

Similarly, nuclear extracts were obtained from BV-2 microglia, which were treated with nimbolide (125, 250 and 500 nM) for 30 min prior to stimulation with LPS (100 ng/ml) for 1 h. Extracts were then used to evaluate DNA binding of NF-κB with a transcription factor assay kit (Abcam), which has a double-stranded DNA sequence containing the NF-κB response element (5′-GGGACTTTCC-3′) immobilised onto the bottom of the wells of a 96-well plate.

### Transient Transfection and Reporter Gene Assays

To determine the effect of nimbolide on the transactivation of NF-κB, a luciferase reporter gene assay was carried out. Cultured BV-2 cells at 60% confluence were transfected with Cignal NF-κB luciferase reporter (Qiagen) using magnetofection (OZ Biosciences), as earlier described [40] and incubated for 20 h at 37°C in a 5% CO_2_ incubator. At the end of the incubation, cells were stimulated with LPS (100 ng/ml) in the presence or absence of nimbolide (125, 250 and 500 nM) for 6 h. Luminescence was measured using POLARstar Optima microplate reader (BMG Labtech). Similar procedures were used in BV-2 cells transfected with Cignal Nrf2 luciferase reporter (Qiagen) and treated with nimbolide (125, 250 and 500 nM) for 24 h. Luciferase activities were evaluated with a Dual-Glo luciferase assay kit (Promega, UK).

### Small Interfering RNA-Mediated Knockdown of Nrf2 and SIRT-1

BV-2 microglia were seeded into 6-well plates at a concentration of 4 × 10^4^ cells/ml and cultured until they were 60% confluence. This was followed by incubation in Opti-MEM for 2 h at 37°C. Complexes containing Glial-Mag transfection reagent (1.8 μl; OZ Biosciences) and either Nrf2 (2 μl; Santa Cruz Biotechnology), SIRT1 (2 μl; Santa Cruz Biotechnology), or control siRNA (Santa Cruz Biotechnology) were then added to the cells. The culture plate was placed on a magnetic plate (OZ Biosciences) for 30 min, followed by incubation at 37 °C for a further 24-h period. Effects of Nrf2 or SIRT1 knockdown on the anti-inflammatory effect of nimbolide were evaluated by treating cells with nimbolide (500 nM) followed by stimulation with LPS (100 ng/ml) for 24 h. Culture supernatants were collected and analysed for levels of nitrite using Griess assay, and TNFα levels were determined using mouse ELISA kits (Biolegend).

### Statistical Analysis

All experiments were carried out at least 3 times. Experimental data are presented as mean ± SEM. Results were analysed with a one-way analysis of variance followed by a post hoc Tukey multiple comparisons test. Results were significant at *p*<0.05. Statistical analyses were done with GraphPad Prism Software version 9.

## Results

### Treatment with Nimbolide Did Not Affect the Viability of LPS-Stimulated BV-2 Microglia

**Fig. 2:**
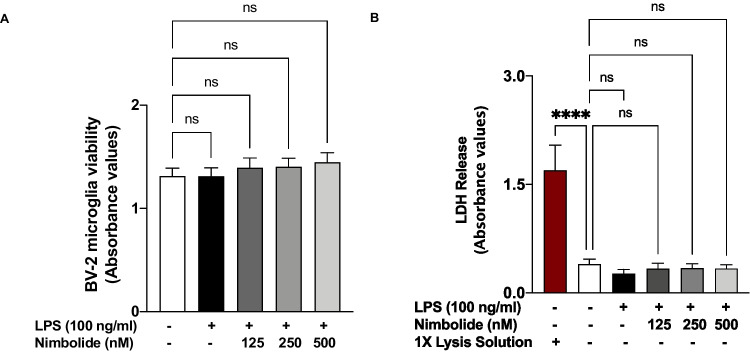
Nimbolide did not affect BV-2 cell viability. XTT assay results showed that there was no significant difference in the viability of LPS-stimulated BV-2 cells treated with nimbolide (125, 250 and 500 nM) when compared with untreated cells (**A**). There was no significant difference in LDH released from BV-2 microglia treated with nimbolide (125, 250 and 500 nM) when compared with control (untreated) cells (**B**). Data are expressed as mean ± SEM for at least three independent experiments. Statistical analysis was performed using one-way ANOVA with post hoc Tukey test (multiple comparisons) ns (not significant), ****p<0.0001, *versus* cell lysis control

XTT cell viability assays to evaluate the effects of 125, 250 and 500 nM concentrations of nimbolide on the viability of LPS-stimulated BV-2 microglia revealed no cytotoxicity following 24 h incubation (Fig. 2A). Similarly, there was no significant difference in LDH release in LPS-stimulated BV-2 cells treated with nimbolide (125, 250 and 500 nM), in comparison with control (untreated and unstimulated) cells (Fig. 2B).

### Effects of Nimbolide on the Production of Pro- and Anti-inflammatory Cytokines

**Fig. 3:**
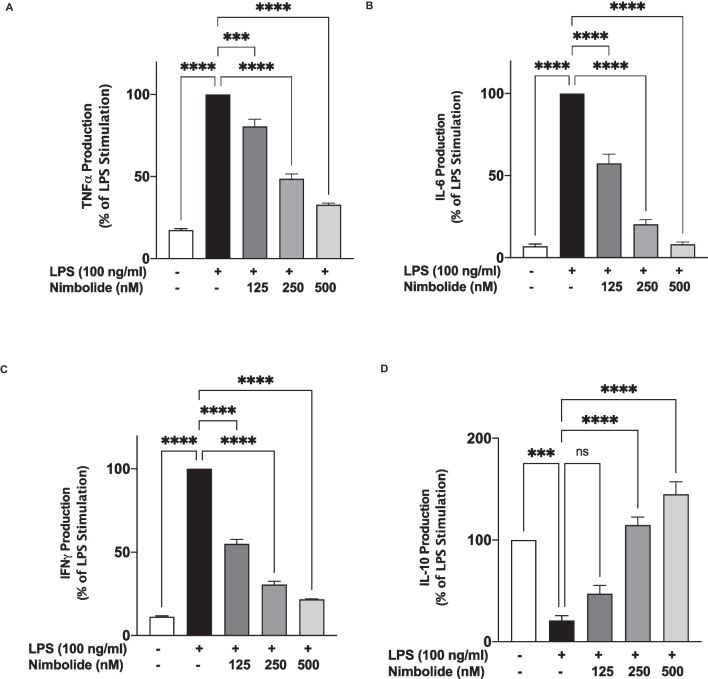
Nimbolide pre-treatment reduced TNFα (**A**), IL-6 (**B**) and IFN-γ (**C**) production, while increasing the release of IL-10 (**D**) in LPS-activated BV-2 microglia. BV-2 cells were pre-treated with nimbolide (125, 250 and 500 nM) prior to stimulation with LPS (100 ng/ml) for 24 h. Supernatants were analysed using mouse ELISA kits. Data are expressed as mean ± SEM for at least 3 independent experiments. Statistical analysis was performed using one-way ANOVA with post hoc Tukey test (multiple comparisons) ns (not significant), ***p<0.001, ****p<0.0001, *versus* LPS control

Following incubation of BV-2 microglia with LPS (100 ng/ml), there were significant (*p*<0.0001) increases in the secretion of pro-inflammatory cytokines TNFα (Fig. 3A), IL-6 (Fig. 3B) and IFNg (Fig. 3C). However, pre-treating cells with nimbolide (125, 250 and 500 nM) resulted in significant (*p*<0.0001) and concentration-dependent reduction in the production of TNFα, IL-6 and IFNγ in these cells. Furthermore, stimulation of BV-2 cells caused a significant (*p*<0.001) suppression in the production of anti-inflammatory cytokine IL-10 (Fig. 3D). In the presence of nimbolide (125 nM), an increase in IL-10 was observed compared with LPS stimulation. However, this increase was not statistically significant. On increasing the concentration of nimbolide to 250 and 500 nM, there were significant (*p*<0.0001) increases in the production of IL-10 in comparison with LPS-stimulated cells (Fig. 3D).

### Nimbolide Reduced iNOS-Mediated LPS-Induced Increased Secretion of NO

**Fig. 4:**
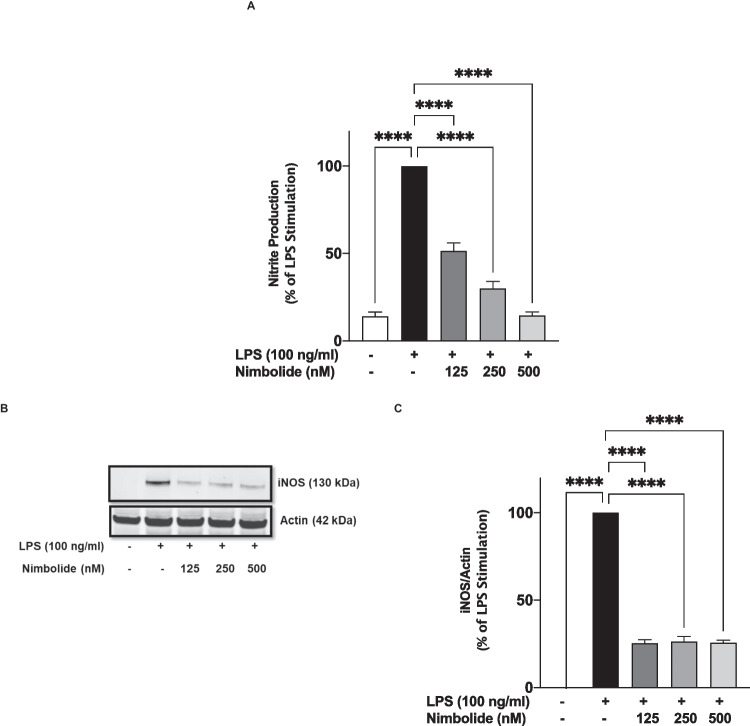
Nimbolide decreased the release of nitric oxide (NO) through the suppression of inducible nitric oxide (iNOS) protein expression in BV-2 microglia stimulated with LPS. BV-2 cells were stimulated with LPS (100 ng/ml) in the presence or absence of nimbolide (125, 250 and 500 nM) for 24 h. Culture supernatants and cell lysates were collected to evaluate levels of nitrite using Griess assay (**A**) and iNOS protein using immunoblotting (**B**) respectively. Densitometric analysis of iNOS immunoblotting (**C**). Data are expressed as mean ± SEM for at least three independent experiments. Statistical analysis was performed using one-way ANOVA with post hoc Tukey test (multiple comparisons). ****p<0.0001, *versus* LPS control

Further investigations on the effects of nimbolide on pro-inflammatory mediator production revealed a significant (*p*<0.001) and concentration-dependent reduction in the production of nitric oxide following pre-treatment with 125, 250 and 500 nM of the compound prior to stimulation of BV-2 microglia with LPS (Fig. 4A). Immunoblotting of lysates obtained from the cells showed a significant reduction in the expression of iNOS protein (Fig. 4B, C).

### Suppressive Effects of Nimbolide on LPS-Induced Increased PGE_2_ Production is Mediated Through COX-2

**Fig. 5:**
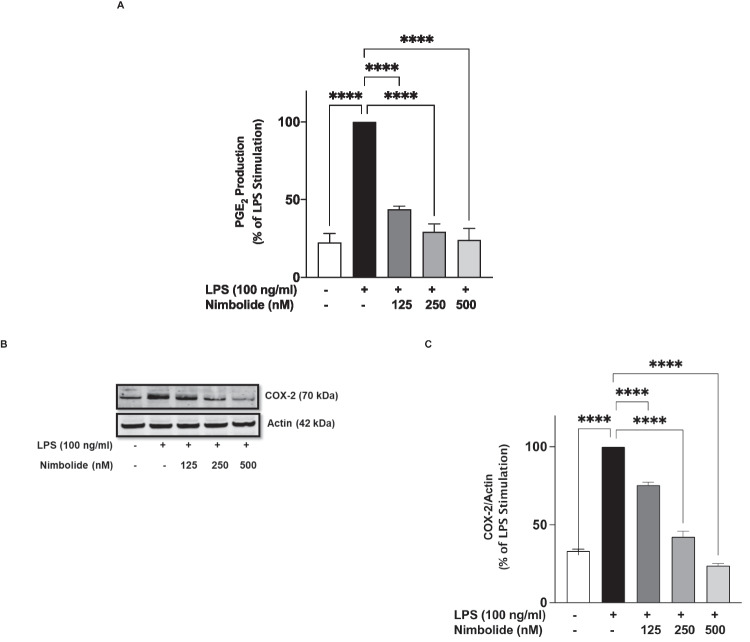
Enzyme immunoassay showing reduction of PGE_2_ production (**A**) and immunoblotting showing inhibition of COX-2 protein expression (**B**) by nimbolide (125, 250 and 500 nM) in LPS-activated BV-2 microglia. Densitometric analysis of COX-2 immunoblotting (**C**). Cells were pre-treated with nimbolide (125, 250 and 500 nM) followed by stimulation of LPS (100 ng/ml) for 24 h. Data are expressed as mean ± SEM for at least three independent experiments. Statistical analysis was performed using one-way ANOVA with post hoc Tukey test (multiple comparisons). ****p<0.0001 *versus* LPS control

Incubation of BV-2 microglia with LPS (100 ng/ml) for 24 h resulted in a ~4.5-fold increase in the secretion of PGE_2_ (Fig. 5A). However, in the presence of nimbolide (125 nM), LPS-induced elevated PGE_2_ production was significantly (*p*<0.0001) reduced from 100 to ~44%. On increasing the concentrations of nimbolide to 250 and 500 nM, PGE_2_ production was further reduced to 29% and 24%, respectively (Fig. 5A). Further studies on cell lysates revealed a concentration-dependent reduction in LPS-induced increased COX-2 protein expression as a result of pre-treatment with 125, 250 and 500 nM nimbolide (Fig. 5B, C).

### Inhibition of Neuroinflammation by Nimbolide is Mediated by Targeting Microglia NF-κB Activation Pathway

**Fig. 6:**
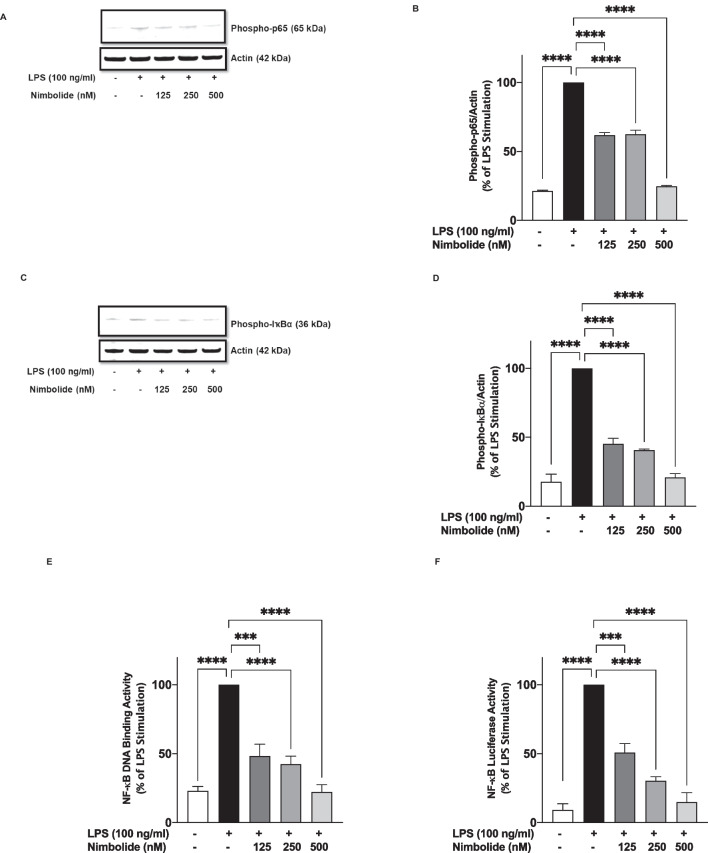
Nimbolide inhibits NF-kκB activation in BV-2 microglia. Nimbolide (125, 250 and 500 nM) reduced levels of phospho-p65 (**A**, **B**) and phospho-IκBα (**C**, **D**) in lysates of BV-2 microglia stimulated with LPS (100 ng/ml). **E** Reduction in LPS-induced increased binding of p65 NF-κB to consensus sites by nimbolide. BV-2 cells were treated with nimbolide (125, 250 and 500 nM) then stimulated with LPS (100 ng/ml) for 30 min and NF-κB transcription factor assay was carried out on nuclear extracts. **F** Nimbolide inhibited LPS-induced NF-κB-dependent gene expression in BV-2 cells. Transfected cells were treated with nimbolide (125, 250 and 500 nM) and stimulated with LPS (100 ng/ml) for 6 h, followed by luciferase reporter gene assay. Data are expressed as mean ± SEM for at least three independent experiments. Statistical analysis was performed using one-way ANOVA with post hoc Tukey test (multiple comparisons). ***p<0.001, ****p<0.0001 *versus* LPS control

Based on our results showing that nimbolide reduced the production of pro-inflammatory mediators in LPS-activated BV-2 microglia, we proceeded to determine whether these effects were mediated through inhibition of NF-κB activation. Immunoblotting analyses of cytoplasmic lysates from LPS-stimulated BV-2 microglia revealed a significant elevation in the expression of phospho-p65 NF-κB protein. Pre-treating the cells with nimbolide (125 and 250 nM) prior to LPS stimulation resulted in an equivalent but significant (*p*<0.0001) reduction in the levels of phospho-p65. However, on increasing the concentration of nimbolide to 500 nM, phospho-p65 was further reduced by ~76%, in comparison with LPS stimulation alone (Fig. 6A, B).

Similar results were obtained when lysates were analysed for protein levels of phospho-IκBα. Stimulation of BV-2 microglia with LPS (100 ng/ml) resulted in an increase in significant (*p*<0.001) increase in phospho-IκBα expression, in comparison with control (unstimulated) cells.

Pre-treatment with nimbolide (125 and 250 nM) prior to LPS stimulation resulted in reduction of phospho-IκBα expression by~55% and ~60%, respectively, in comparison to LPS stimulation alone. Furthermore, increasing the concentration of nimbolide to 500 nM caused a reduction in phospho-IkBa protein expression by 79%, when compared with LPS stimulation (Fig. 6C, D).

Assessment of nuclear events revealed that following a 60-min stimulation of BV-2 microglia, there was a significant (*p*<0.001) increase in the binding of NF-κB to consensus sites in the nucleus (Fig. 6E). In the presence of nimbolide (125, 250 and 500 nM), there was a concentration-dependent reduction in the DNA binding of NF-κB (Fig. 6E).

Furthermore, reporter gene assays were conducted to evaluate the effects of nimbolide on the ability of NF-κB to activate the expression of genes encoding pro-inflammatory proteins such as the cytokines, iNOS and COX-2. LPS stimulation of BV-2 cells transfected with NF-κB luciferase reporter plasmid resulted in marked luciferase activity, as shown in Fig. 6 F. On pre-treating cells with nimbolide (125, 250 and 500 nM), concentration-dependent inhibition of NF-κB luciferase activity was observed (Fig. 6F).

### Nimbolide Induced Deacetylation of p65 in LPS-Activated Microglia

**Fig. 7:**
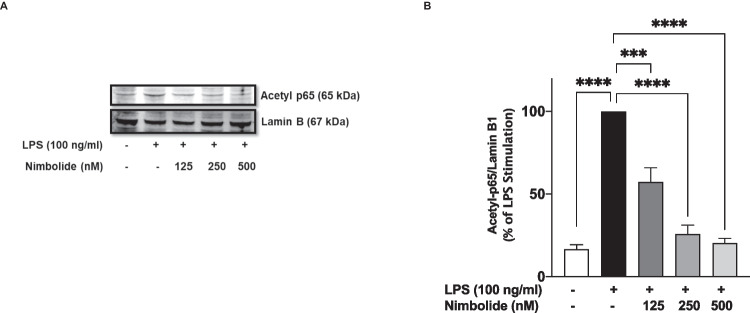
Nimbolide interferes with acetylation of p65 sub-unit following activation with LPS. Nimbolide-treated BV-2 microglia were stimulated with LPS (100 ng/ml). Western blotting on nuclear extracts showed reduction in protein expression of acetyl-p65 (**A**). Densitometric analysis of blots (**B**). Densitometric values are expressed as mean ± SEM for at least three independent experiments. Statistical analysis was performed using one-way ANOVA with post hoc Tukey test (multiple comparisons). ***p<0.001, ****p<0.0001 *versus* LPS control

Acetylation of lysine residues in NF-κB p65 has been shown to determine its activation or deactivation. Expectedly, LPS stimulation of BV-2 cells resulted in increased expression of acetyl-p65 protein (lysine 310) (Fig. 7A, B). In the presence of nimbolide (125, 250 and 500 nM), a concentration-dependent reduction in acetylation of NF-κB p65.

### Nimbolide Reduced Protein Levels of Phospho-p38 and Phospho-JNK in LPS-Stimulated BV-2 Microglia

It is known that the production of some pro-inflammatory cytokines following inflammatory stimuli is partly linked to the activation of MAPKs, especially p38 and JNK. Experiments were therefore carried out to investigate whether nimbolide could affect the activation of these proteins.

**Fig. 8:**
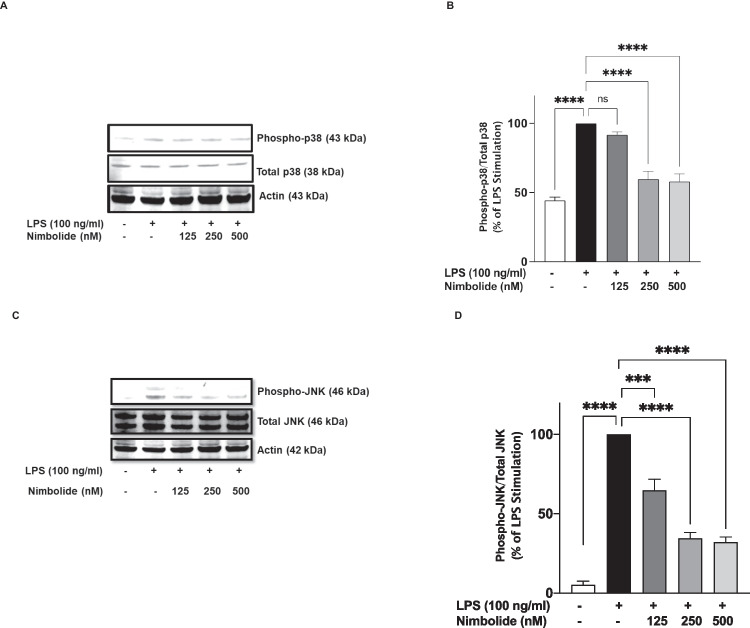
Nimbolide targets MAPK activation in BV-2 microglia. Cells were treated with nimbolide (125, 250 and 500 nM) prior to stimulation with LPS (100 ng/ml). Immunoblot analyses shows reduction in protein expression of phospho-p38 (**A**, **B**), and phospho-JNK (**C**, **D**). Densitometric values are expressed as mean ± SEM for at least three independent experiments. Statistical analysis was performed using one-way ANOVA with post hoc Tukey test (multiple comparisons). ns (not significant) ***p<0.001, ****p<0.0001 *versus* LPS control

Incubation of BV-2 microglia with LPS (100 ng/ml) caused an increase in protein expression of phospho-p38α, indicating activation. In the presence of 125 nM nimbolide, there was no significant (*p*<0.05) reduction in LPS-induced increase in phospho-p38α protein (Fig. 8A, B). On increasing the concentration of nimbolide to 250 and 500 nM, there was a significant (*p*<0.001) reduction in phospho-p38, in comparison with LPS stimulation alone (Fig. 8A, B). Similarly, pre-treatment of BV-2 microglia with nimbolide (125, 250 and 500 nM) prior to LPS stimulation resulted in a significant reduction in phospho-JNK protein, when compared with LPS stimulation alone (Fig. 8C, D).

### Effects of Nimbolide on Cellular ROS Generation and Protein Levels of (NOX2) gp91^phox^

**Fig. 9:**
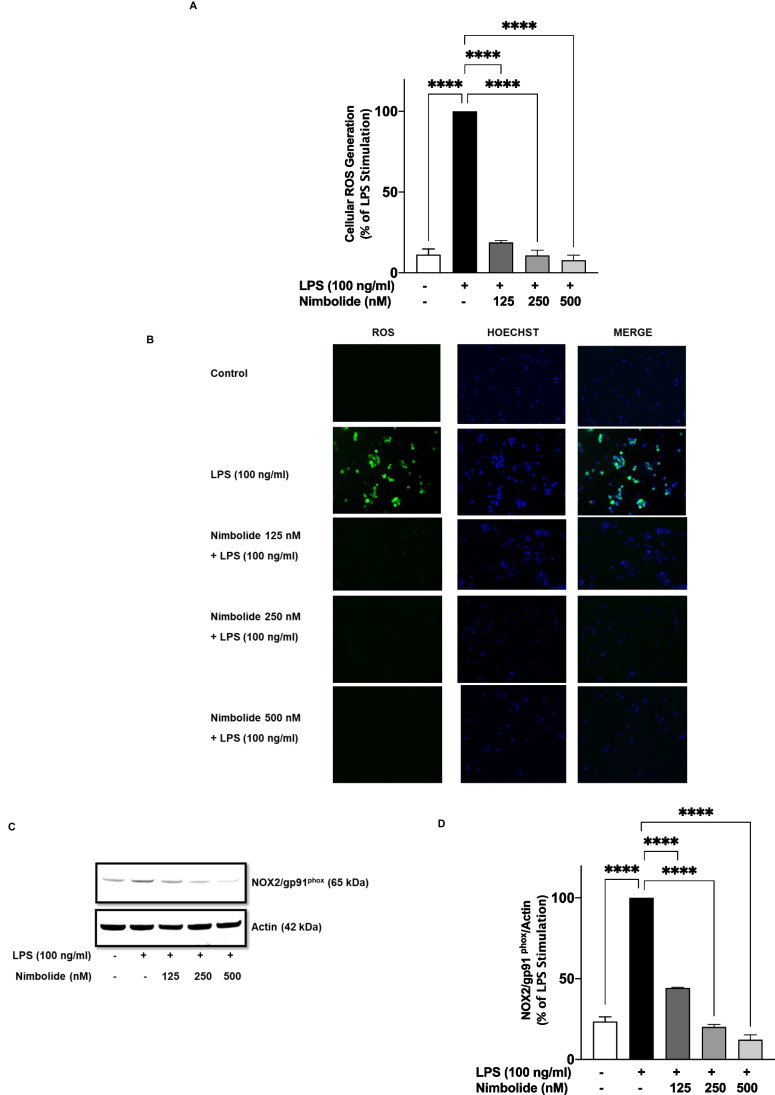
Nimbolide inhibited LPS-induced cellular ROS generation and gp91^phox^ protein expression in BV-2 microglia. Cells were stained with DCFDA for 45 min at 37°C, then stimulated with LPS (100 ng/ml) with or without nimbolide (125, 250 and 500 nM) for 24 h. Fluorescence detection of ROS generation was done using Polar star Optima plate reader (**A**) and EVOS® Floid® cell imaging station (**B**). Stimulation of BV-2 microglia resulted in elevated protein expression of NOX2/gp91^phox^, which was significantly reduced by nimbolide (**C**, **D**). Data are expressed as mean ± SEM for at least three independent experiments. Statistical analysis was performed using one-way ANOVA with post hoc Tukey test (multiple comparisons). ****p<0.0001 versus LPS control

Results of experiments to evaluate the effects of nimbolide on the cellular generation of ROS following stimulation with LPS are shown in Fig. 9 A and B. Microplate detection of ROS revealed that stimulation of BV-2 microglia with LPS (100 ng/ml) resulted in ~90% increase in ROS generation, an outcome that was markedly diminished in the presence of 125, 250 and 500 nM nimbolide (Fig. 9A). These effects were confirmed using fluorescence microscopy, which revealed a marked reduction in DCFDA fluorescence by LPS-activated microglia pre-treated with 125 nM nimbolide. In contrast, 250 and 500 nM concentrations of the compound produced almost complete inhibition of fluorescence (Fig. 9B).

In the presence of pro-inflammatory stimuli such as LPS, an increase in the expression of gp91^phox^ protein results in cellular generation of ROS. Based on results showing the inhibitory effects of nimbolide on LPS-induced increased ROS production, the effects of the compound on protein levels of gp91phox were also evaluated. Results in Fig. 9 C and D show an increase in the expression of gp91^phox^ protein following stimulation of BV-2 microglia with LPS. However, in the presence of nimbolide (125, 250 and 500 nM), a concentration-dependent and significant (*p*<0.001) reduction in the levels of gp91^phox^ protein was observed.

### Nimbolide Directly Increased Protein Levels of HO-1 and NQO-1 Antioxidant Proteins in BV-2 Microglia

**Fig. 10:**
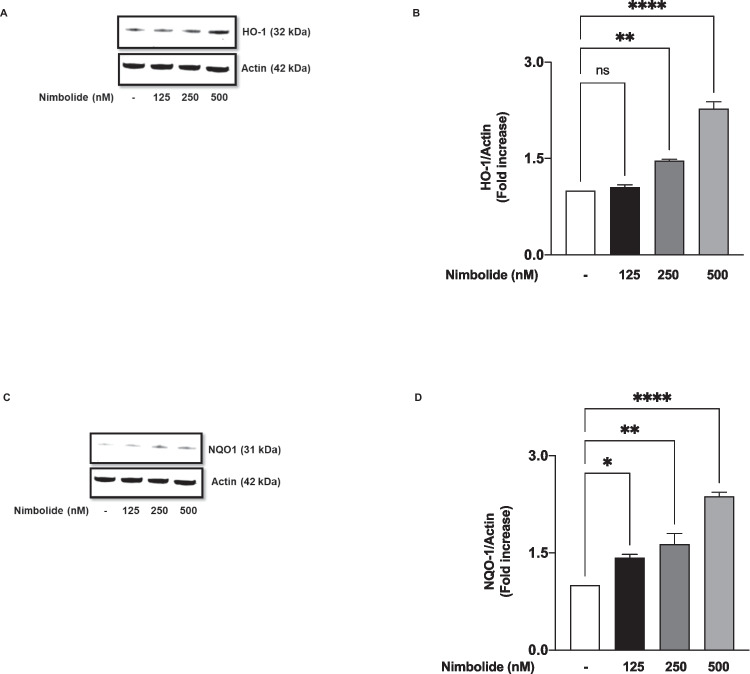
Nimbolide increased protein levels of HO-1 (**A**, **B**) and NQO-1 (**C**, **D**) in BV-2 microglia. Cells were treated with nimbolide (125, 250 and 500 nM) for . Thereafter, lysates were analysed using immunoblotting. Densitometric data are expressed as mean ± SEM for at least three independent experiments. Statistical analysis was performed using one-way ANOVA with post hoc Tukey test (multiple comparisons). ns (not significant), *p<0.05, **p<0.01, ****p<0.0001, *versus* untreated control

Following results showing the antioxidant activity of nimbolide through a gp91^phox^-mediated reduction in ROS generation in LPS-stimulated microglia, further experiments were conducted to determine the direct effects of the compound on levels of HO-1 and NQO-1 antioxidant proteins in the cells. Incubation of BV-2 microglia with 125 nM nimbolide did not affect basal levels of HO-1 (~1.05-fold increase). Nevertheless, increasing the concentration of the compound to 250 and 500 nM caused ~1.5- and ~2.3-fold increases in the expression of HO-1 (Fig. 10A, B). Interestingly, incubating the cells with 125 nM of nimbolide resulted in a significant (*p*<0.05) ~1.4-fold increase in NQO-1 protein expression. In the presence of higher concentrations (250 and 500 nM) of the compound, there were ~1.6- and ~2.4-fold increases in NQO-1 protein levels, respectively (Fig. 10C, 10D).

### Nimbolide Directly Activates Nrf2

Nrf2 is a transcription factor that regulates antioxidant responses through the expression of genes encoding proteins such as HO-1 and NQO-1. Encouraged by the results demonstrating an increase in the expression of these proteins by nimbolide, experiments were conducted to determine the effects of the compound on mechanisms involved in the activation of Nrf2.

**Fig. 11:**
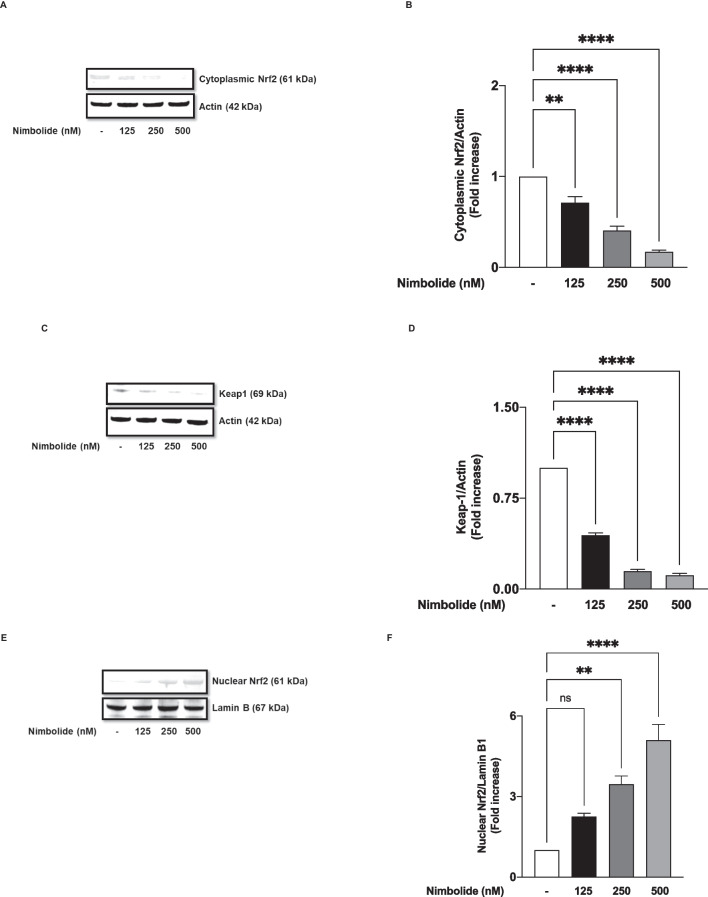
Nimbolide activates Nrf2 antioxidant mechanisms in BV-2 microglia. Cells were treated with nimbolide (125, 250 and 500 nM) for 24 h. Immunoblotting of cytoplasmic extracts revealed disappearance of Nrf2 (**A**, **B**) and Keap-1 (**C**, **D**). Results of western blotting of nuclear extracts showing accumulation of Nrf2 (**E**, **F**). Densitometric values are expressed as mean ± SEM for at least three independent experiments. Statistical analysis was performed using one-way ANOVA with post hoc Tukey test (multiple comparisons). ns (not significant), **p<0.01, ****p<0.0001, *versus* untreated control

Results in Fig. 11 A and B show that treating BV-2 microglia with nimbolide (125, 250 and 500 nM) resulted in concentration-dependent and significant (*p*<0.01) decrease in protein levels of cytoplasmic Nrf2, accompanied by corresponding concentration-dependent and significant (*p*<0.001) reduction in Keap-1 protein (Fig. 11C, D). These outcomes indicate that nimbolide treatment induced translocation of Nrf2 from the cytoplasm due to the degradation of Keap-1.

**Fig. 12:**
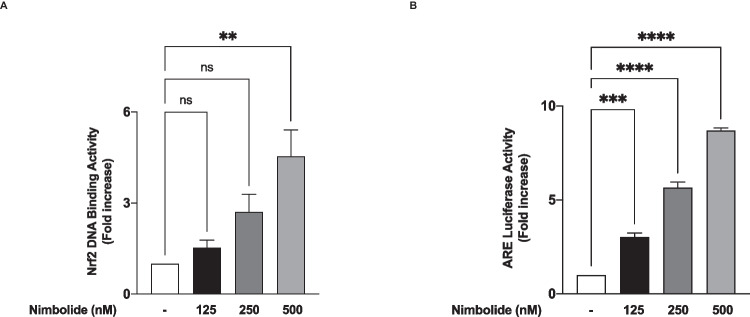
Nimbolide activates Nrf2 antioxidant mechanisms in BV-2 microglia. Nuclear extracts from BV-2 cells were added to 96-well plates to which an oligonucleotide containing the ARE consensus binding site (5′ GTCACAGTGACTCAGCAGAATCTG-3′) has been immobilised, followed by addition of Nrf2 and HRP-conjugated antibodies. Absorbance was read in a microplate reader (**A**). Effects of nimbolide on ARE luciferase activity in BV-2 microglia (**B**). Data are expressed as mean ± SEM for at least three independent experiments. Statistical analysis was performed using one-way ANOVA with post hoc Tukey test (multiple comparisons). ns (not significant), **p<0.01, ***p<0.001, ****p<0.0001, *versus* untreated control

In the presence of nimbolide (125 nM), there was an insignificant (*p*<0.05) increase in the expression of Nrf2 in the nucleus when compared with untreated cells. However, on treating the cells with 250 and 500 nM of the compound, a significant (*p*<0.01) increase in nuclear expression of Nrf2 was observed (Fig. 11E, F). Transcription factor assays revealed that a significant (*p*<0.01) increase in binding to consensus sites was achieved with 500 nM of nimbolide, while the increase observed with lower concentrations of the compound was not significant (*p*<0.05) (Fig. 12A). Results in Fig. 11 B show that nimbolide treatment produced significant (*p*<0.001) and concentration-dependent elevation in ARE luciferase activity, suggesting an increase in the ability of the compound to produce a direct increase in the ability of Nrf2 to activate the expression of genes encoding antioxidant proteins.

### Nrf2 is Required for the Anti-inflammatory Activity of Nimbolide in BV-2 Microglia

**Fig. 13:**
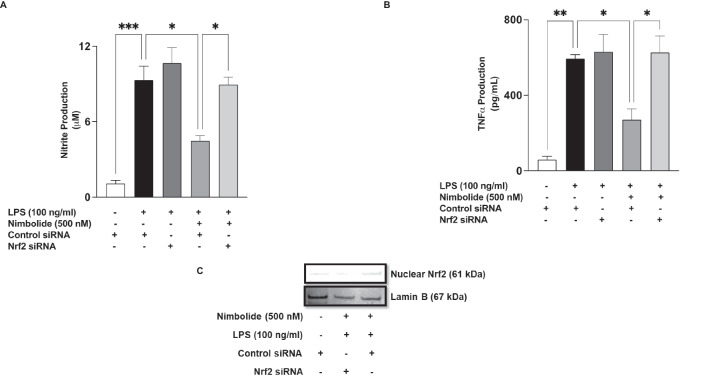
Effects of Nrf2 knockdown on anti-inflammatory effects of nimbolide. Control siRNA- and Nrf2 siRNA-transfected BV-2 cells were pre-treated with nimbolide (500 nM) prior to stimulation with LPS (100 ng/ml) for 24 h. Culture supernatants were analysed for nitrite (**A**) and TNFα (**B**). Western blot experiments on nuclear extracts to determine knockout efficiency (**C**). Data are expressed as mean ± SEM for at least three independent experiments. Statistical analysis was performed using one-way ANOVA with post hoc Tukey test (multiple comparisons). *p<0.05, **p<0.01, ***p<0.001, nimbolide + LPS in Nrf2 siRNA-transfected cells *versus* nimbolide + LPS in control siRNA-transfected cells

Following the observation that nimbolide produced a direct enhancement of Nrf2 activation in BV-2 microglia, experiments were conducted to explore the role of Nrf2 induction in the anti-inflammatory activity of the compound. Results in Fig. 13 A show that in cells transfected with control siRNA, significant (*p*<0.001) LPS-induced elevation in NO production was reduced (*p*<0.05) by nimbolide (500 nM) pre-treatment. On the other hand, in Nrf2 siRNA-transfected cells, pre-treatment with nimbolide prior to stimulation with LPS did not result in a significant (*p*<0.05) reduction in NO production. Similar results (Fig. 13B) were observed in assays to determine TNFα production. siRNA knockdown of Nrf2 in BV-2 cells resulted in the loss of TNFα suppressive ability of nimbolide (500 nM). Western blotting experiments to evaluate transfection efficiency showed reduced nuclear Nrf2 expression in nuclear extracts of cells incubated with Nrf2 siRNA when compared with extracts from cells incubated with control siRNA (Fig. 13C).

### Nimbolide Increased Nuclear SIRT-1 Protein

**Fig. 14:**
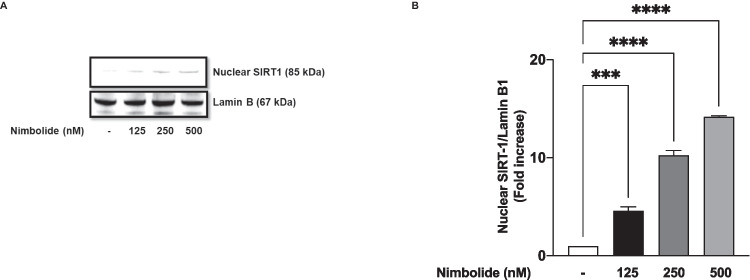
Nimbolide activates SIRT-1 in BV-2 microglia. BV-2 cells were treated with nimbolide, followed by immunoblotting of nuclear extracts (**A**). Densitometric analysis of blots (**B**). Densitometric values are expressed as mean ± SEM for at least three independent experiments. Statistical analysis was performed using one-way ANOVA with post hoc Tukey test (multiple comparisons). ***p<0.001, ****p<0.0001 *versus* untreated control

**Fig. 15:**
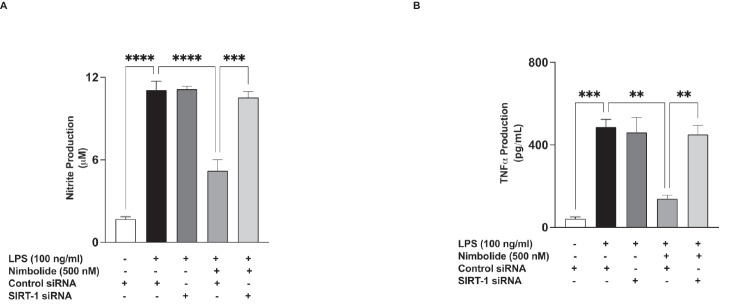
Effects of SIRT-1 knockdown on anti-inflammatory effects of nimbolide. Control siRNA- and SIRT-1 siRNA-transfected BV-2 cells were pre-treated with nimbolide (500 nM) prior to stimulation with LPS (100 ng/ml) for 24 h. Culture supernatants were analysed for nitrite (**A**) and TNFα (**B**). Data are expressed as mean ± SEM for at least three independent experiments. Statistical analysis was performed using one-way ANOVA with post hoc Tukey test (multiple comparisons). *p<0.05, **p<0.01, ***p<0.001, nimbolide + LPS in SIRT-1 siRNA-transfected cells *versus* nimbolide + LPS in control siRNA-transfected cells

Studies have linked the deacetylation of NF-κB p65 to nuclear SIRT-1, and results from this study have shown that nimbolide prevented LPS-induced acetylation of NF-κB p65 at lysine 310. Interestingly, western blot analyses revealed that incubation of BV-2 microglia with nimbolide (125, 250 and 500 nM) resulted in significant (*p*<0.001) and concentration-dependent increase in nuclear SIRT-1 protein expression (Fig. 14A, B). These results prompted experiments to determine if nimbolide-induced increase in SIRT-1 protein in the nucleus may contribute to its anti-inflammatory activities. Results of siRNA experiments in Fig. 15 A and B show that siRNA-mediated knockdown of SIRT-1 resulted in the loss of anti-inflammatory activity of nimbolide as measured by the production of NO and TNFα.

## Discussion

Nimbolide is a compound found in *Azadiractha indica*, a plant that has been linked with in vivo and in vitro anti-inflammatory effects [41–45]. Earlier, nimbolide was shown to reduce the expression of pro-inflammatory cytokines IL-6, IL-8, IL-12 and TNFα in LPS-stimulated RAW 264.7 and IL-10^−/−^ peritoneal macrophages [39]. In a separate study, nimbolide reduced the levels of pro-inflammatory cytokines (IL-1β, IL-6, IL-12, TNFα, TGFβ) and chemokines (MIP-1α and MIP-1β), while increasing levels of anti-inflammatory cytokines (IL-4, IL-10 and IL-13) in lung tissues of mice and in RAW 264.7 and A549 cells [46]. In this study, we showed that nimbolide inhibited neuroinflammation through the reduction of LPS-induced increased production of pro-inflammatory cytokines TNFα, IL-6 and IFNg, as well as increasing production of IL-10.

Inducible nitric oxide synthase–mediated excessive production of NO in microglia significantly contributes to neuron-damaging neuroinflammation in response to agents like lipopolysaccharide and cytokines. Consequently, targeting iNOS-mediated NO production is a critical strategy in neuroinflammation. This study found that inhibition of iNOS protein, accompanied by suppression of NO production in LPS-activated BV-2 microglia, contributes to the inhibition of neuroinflammation by nimbolide. Notably, this compound has shown similar profiles in RAW 264.7 macrophages stimulated with LPS and in cerulein-induced pancreatic inflammation in mice [46, 47]. In this study, inhibition of neuroinflammation by nimbolide was also evidenced by a reduction in protein expression of COX-2, with an accompanying reduction in the secretion of PGE_2_.

Nimbolide belongs to a chemical group of natural products known as terpenoids, a structurally diverse group of compounds [48] which have been widely reported to be valuable as inhibitors of neuroinflammation in vivo and in vitro [49]. Examples of terpenoids which have been shown to produce similar anti-neuroinflammatory effects include artemisinin [50], carnosol [51], carnosic acid [52], parthenolide [53] and thymoquinone [54].

The NF-κB transcription factor promotes neuroinflammation through positive regulation of genes encoding pro-inflammatory cytokines such as TNFα and IL-6, as well as iNOS and COX-2. Since nimbolide reduced the production of some pro-inflammatory mediators, which are known to be under the regulatory control of NF-κB, investigations were carried out to determine whether the compound produced anti-inflammatory activity in the microglia by targeting the NF-κB activation pathway. Activation of this pathway involves phosphorylation of the IκBα-NF-κB p65 complex, followed by proteasomal degradation of IκBα and translocation of the p65 subunit to the nucleus [55, 56]. This study found that nimbolide blocks phosphorylation of IκBα and NF-κB p65 sub-units, which suggests that the compound may be acting in the cytoplasm to target events resulting in the translocation of NF-κB to the nucleus. However, this study did not establish whether nimbolide could act on upstream targets involving LPS-activated toll-like receptor (TLR) adapters, MyD88 and TRIF.

Following release from the cytoplasm, free NF-κB binds to promoter and enhancer regions containing κB sites in the nucleus. Interestingly, transcription factor assay results showed that nimbolide prevented the binding of NF-κB to GGGACTTTCC consensus sites in LPS-activated BV-2 microglia. The impact of nimbolide on the DNA binding capacity of NF-κB is probably a reflection of its action in the cytoplasm by preventing the translocation of the transcription factor to the nucleus. This effect may also account for the diminished transactivation of NF-κB observed in LPS-stimulated microglia treated with nimbolide.

Similar NF-κB inhibitory activity has been reported for nimbolide in LPS-stimulated A549 cells and lung tissues [46], as well as in studies employing intestinal epithelial cells and macrophages [39]. Inhibition of NF-κB activation by nimbolide in BV-2 microglia reported in this study suggests that the compound targets the transcription factor in peripheral and CNS immune cells and non-immune cells such as epithelial cells.

The MAPKs mediate a variety of cellular responses. However, p38 MAPK has been linked to microglia activation and neuroinflammation [13–16]. Similarly, JNK has been shown to phosphorylate c-Jun and ATF-2 proteins resulting in increased transcriptional activity of the AP-1 and, thus, upregulation of cytokine production [57]. Results of this study showed that nimbolide inactivated both p38 and JNK MAPKs through inhibition of their phosphorylation. The observed effects on these MAPKs suggest that their inhibition by nimbolide may in fact contribute to its ability to reduce the production of pro-inflammatory mediators. This proposal is justified by reports linking the activation of p38 to mRNA stability of COX-2, TNF-α, IL-6 and iNOS genes [57, 58].

The binding of LPS to TLR4 activates the MyD88-dependent activation of TRAF-6, which in turn catalyses the phosphorylation of TAK-1. Activated TAK-1 then initiates phosphorylation processes resulting in the activation of NF-κB and MAPK pathways [59]. Dual inhibition of LPS-induced NF-κB and MAPK activation by nimbolide could be explained by the potential targeting of one or more of these upstream signals.

In the microglia, NADPH oxidase (NOX) is a group of five enzymes which catalyse the generation of ROS. Specifically, NOX2 (gp91^phox^) is highly expressed in the microglia [60]. Reports of several studies have indicated that NOX-mediated generation of ROS in microglia is linked to both NF-κB and MAPK activation [61]. In this study, we showed that LPS-induced increased ROS generation and gp91^phox^ protein levels in BV-2 microglia were reduced in the presence of nimbolide, which suggests that this compound could be promoting anti-inflammatory effects through mechanisms involving inhibition of oxidative stress.

Nrf2 is a transcription factor involved in redox signalling, as well as in antioxidant and anti-inflammatory responses. Under physiological conditions, Nrf2 is complexed in the cytoplasm by Keap1. However, in response to oxidative stress, Nrf2 dissociates from Keap-1 and translocates into the nucleus to activate the transcription of antioxidant genes. Results showing that nimbolide reduced LPS-induced increased oxidative stress in the microglia prompted an investigation which revealed that the compound increased levels of antioxidant proteins (HO-1 and NQO-1) while activating Nrf2 through direct actions on binding to antioxidant response elements (ARE) in the nucleus. These findings reflect studies reported by Rojo et al. (2010, 2018), who showed that Nrf2 knockout in mice resulted in an increase in microglia activation accompanied by elevated levels of COX-2, iNOS, IL-6 and TNFα [20, 21]. RNAi was used to show further a loss of anti-inflammatory activity by nimbolide following knockdown of Nrf2. These are interesting results that suggest that nimbolide inhibits neuroinflammation and oxidative stress through its ability to enhance Nrf2 activation and induce an antioxidant response.

The sirtuin SIRT1 inhibits NF-κB signalling through the deacetylation of lysine residue 310 on the RelA/p65 subunit [30]. Based on the role of acetylation at 310 in the transcriptional activity of NF-κB [62], we conducted experiments which showed that nimbolide attenuated LPS-induced acetylation of p65 sub-unit in BV-2 microglia. We further showed that treatment of BV-2 microglia with nimbolide resulted in the activation of SIRT-1, as shown by its nuclear accumulation. Furthermore, activation of SIRT-1 appears to contribute to the anti-inflammatory activity of nimbolide as shown by the loss of this activity in SIRT-1 siRNA-transfected cells stimulated with LPS.

The outcome of this study reveals that nimbolide is a natural product which inhibits neuroinflammation in BV-2 microglia through as-yet-unknown mechanisms resulting in dual inhibition of NF-κB and MAPK pathways. We further report that activation of Nrf2 antioxidant mechanisms contributes to its anti-inflammatory activity in BV-2 microglia.

## Data Availability

The datasets generated during and/or analysed during the current study are available from the corresponding author on reasonable request.
